# Silver nanoparticle interactions with glycated and non-glycated human serum albumin mediate toxicity

**DOI:** 10.3389/ftox.2023.1081753

**Published:** 2023-02-28

**Authors:** Hee-Yon Park, Christopher Chung, Madeline K. Eiken, Karl V. Baumgartner, Kira M. Fahy, Kaitlyn Q. Leung, Evangelia Bouzos, Prashanth Asuri, Korin E. Wheeler, Kathryn R. Riley

**Affiliations:** ^1^ Department of Chemistry and Biochemistry, Swarthmore College, Swarthmore, PA, United States; ^2^ Department of Chemistry and Biochemistry, Santa Clara University, Santa Clara, CA, United States; ^3^ Department of Bioengineering, Santa Clara University, Santa Clara, CA, United States

**Keywords:** nanotoxicity, protein corona, silver nanoparticles, glycation, post-translational modification (PMT)

## Abstract

**Introduction:** Biomolecules bind to and transform nanoparticles, mediating their fate in biological systems. Despite over a decade of research into the protein corona, the role of protein modifications in mediating their interaction with nanomaterials remains poorly understood. In this study, we evaluated how glycation of the most abundant blood protein, human serum albumin (HSA), influences the formation of the protein corona on 40 nm silver nanoparticles (AgNPs) and the toxicity of AgNPs to the HepG2 human liver cell line.

**Methods:** The effects of glycation on AgNP-HSA interactions were quantified using circular dichroism spectroscopy to monitor protein structural changes, dynamic light scattering to assess AgNP colloidal stability, zeta potential measurements to measure AgNP surface charge, and UV-vis spectroscopy and capillary electrophoresis (CE) to evaluate protein binding affinity and kinetics. The effect of the protein corona and HSA glycation on the toxicity of AgNPs to HepG2 cells was measured using the WST cell viability assay and AgNP dissolution was measured using linear sweep stripping voltammetry.

**Results and Discussion:** Results from UV-vis and CE analyses suggest that glycation of HSA had little impact on the formation of the AgNP protein corona with protein-AgNP association constants of ≈2x10^7^ M^-1^ for both HSA and glycated HSA (gHSA). The formation of the protein corona itself (regardless of whether it was formed from HSA or glycated HSA) caused an approximate 2-fold decrease in cell viability compared to the no protein AgNP control. While the toxicity of AgNPs to cells is often attributed to dissolved Ag(I), dissolution studies showed that the protein coated AgNPs underwent less dissolution than the no protein control, suggesting that the protein corona facilitated a nanoparticle-specific mechanism of toxicity. Overall, this study highlights the importance of protein coronas in mediating AgNP interactions with HepG2 cells and the need for future work to discern how protein coronas and protein modifications (like glycation) may alter AgNP reactivity to cellular organisms.

## 1 Introduction

The medicinal use of nanoparticles has expanded in the last few decades, including diagnostic, imaging, and therapeutic applications. This growth is driven by the increasing control of the chemical and physical properties of nanoparticles (NPs). In parallel, a mechanistic understanding of cellular and organismal response to NPs has developed. At the molecular level, NPs entering a biological system adsorb a population of biomolecules, including proteins, lipids, sugars, or DNA. This biomolecular coating is dominated by an abundance of proteins, forming a protein corona (PC) (Lynch et al., 2007; Zhang et al., 2018; Griffith et al., 2020; Lima et al., 2020). The PC significantly alters NP physicochemical properties, including surface charge and agglomeration state, as well as biological fate, biodistribution, and toxicity (Walczyk et al., 2010; Fleischer and Payne, 2014; Caracciolo et al., 2017; Shannahan, 2017; Nierenberg et al., 2018; Lima et al., 2020). Although often cited as a barrier to development of nanomedicines, the PC can also be used to increase sensitivity and therapeutic efficacy of NPs (Caracciolo et al., 2017; Corbo et al., 2017; García Vence et al., 2020).

Formation of the PC is mediated by the properties of the NP, as well as the properties of the solution and population of biomolecules within the solution. Biochemical features of the latter may vary across populations and individuals, depending upon physiological conditions and disease states. There is also significant evidence of spatiotemporal changes to the composition of the corona as biomolecules undergo dynamic association and dissociation with the NP surface and with each other over time and as the NP is transported through a biological organism (Cai et al., 2022; Cox et al., 2018; Dell’Orco et al., 2010; Weiss et al., 2019). Thus, PCs formed in serum, around otherwise identical NPs, vary significantly depending upon whether a patient is pregnant, has a cold, or has diabetes, among other medical conditions (Hajipour et al., 2014; Corbo et al., 2017; García Vence et al., 2020; Ju et al., 2020). This personalized PC, in turn, not only dictates the abundance and populations of proteins in the corona, but also results in variations in the zeta-potential of the protein coated NP and its interaction with cells (Hajipour et al., 2014; Ju et al., 2020). Therefore, the personalized PC can be used to extrapolate and identify biomarkers for disease, while serving to improve the safety and efficacy of nanomedicines.

Among the biophysical features of proteins that change across individuals are post-translational modifications (PTMs). Despite the abundance and significance of PTMs, their role at the nano-bio interface remains relatively unexplored. Glycation and glycosylation are among the most abundant PTMs. Glycosylation, which refers to the enzyme-mediated addition of glycans, plays a variety of structural and functional roles—from molecular recognition and cellular signaling to protein trafficking and enzymatic regulation (Varki and Gagneux, 2015). Glycation, on the other hand, refers to the non-enzymatic addition of monosaccharides to proteins. Changes in glycosylation and glycation profiles are associated with a variety of diseases, including cancer and diabetes (Varki and Gagneux, 2015). For example, HbA1c, a common indicator test for diabetes, examines glucose bound to hemoglobin due to glycation and serves as an indicator of long-term blood glucose levels. In parallel, the glycation of another common blood protein, human serum albumin (HSA), can be used similarly. Interestingly, glycation contributes to changes in HSA structure and function, depending on the nature and amount of glycation (Qiu et al., 2021).

While impacts of protein glycation have not been specifically interrogated within PC studies, a few studies have assessed the role of glycosylation on PC formation and NP cellular interactions. Such studies are often motivated by the understanding that glycosylation can mediate cellular uptake of drugs (Cai et al., 2018; Ghazaryan et al., 2019). In the context of PC studies, glycosylation alters the population of proteins in the PC and alters adhesion of NPs to cell membranes (Wan et al., 2015; Ghazaryan et al., 2019; Clemente et al., 2022). More recently, the PC was used to identify enrichment of glycoproteins and identify disease biomarkers (Trinh et al., 2022). In a more focused study of just one commonly glycosylated blood protein, transferrin, significant variations in binding strength were observed with glycosylation, as well as protein structural changes upon interaction with gold NPs (Barbir et al., 2021). With an understanding of the impact of glycans on NP interactions and PC formation, the safety and efficacy of NP-enabled drugs and diagnostics will increase, enabling precision medicine and personalized medicines (Hajipour et al., 2014; Corbo et al., 2017; García Vence et al., 2020).

Due to their antimicrobial properties, silver nanoparticles (AgNPs) are used in a variety of medical conditions, including bandages for long-term wound healing for diabetic wounds (Choudhury et al., 2020). In disease states such as poorly managed diabetes, high blood sugar drives glycation of common blood proteins and may influence PC formation and AgNP behavior. Thus, we aimed to assess the role of glycation on the interaction of the most abundant blood protein, HSA, with AgNPs, and evaluate subsequent impacts on cellular toxicity. In our study, we chose 40 nm AgNPs, which is within the size range of AgNP sizes commonly used in wound dressings (Sussman et al., 2015; Paladini and Pollini, 2019; Krishnan et al., 2020). HSA is glycated under a variety of conditions (Varki and Gagneux, 2017) and has been consistently identified as a component of the AgNP-PC formed from human serum and plasma (del Pilar Chantada-Vázquez et al., 2019; Gorshkov et al., 2019; Lai et al., 2017; Shannahan, 2017). We hypothesized that glycation of HSA would alter interactions with AgNPs, due to changes in the accessible functional groups on HSA, as well as other changes in structure upon glycation. In turn, we also speculated that these structural changes would alter HSA-coated AgNP cellular impacts, as glycation of HSA been shown to alter absorption, distribution, efficacy and excretion of other drugs (Qiu et al., 2021). We expand upon previous studies of HSA and AgNP interactions to first evaluate the role of HSA glycation in AgNP and HSA structural stability and biophysical properties, including AgNP agglomeration, oxidation, and charge state. To evaluate the impacts of AgNPs on protein interactions and structure, binding affinities and protein structures are also compared. Second, we place these biophysical results in the context of downstream impacts by monitoring the toxicity of HSA and gHSA coated AgNPs in a live human liver cell line. Finally, we evaluate the effect of the HSA and gHSA PCs on AgNP dissolution as a first step towards understanding the mechanism by which PCs alter AgNP toxicity.

## 2 Materials and methods

### 2.1 Materials

Sodium citrate monobasic, sodium chloride, Trizma base, glycine, hydrochloric acid, 70% nitric acid, HSA, and glycated HSA (gHSA) were from Sigma-Aldrich (St. Louis, MO). The degree of glycation of gHSA was 3 mol hexose (as fructosamine) per mol albumin and the purity was 94% according to the manufacturer’s certificate of analysis. BioPure citrate stabilized AgNPs were purchased from nanoComposix (San Diego, CA). AgNPs had a nominal diameter of 40 nm and were supplied at a concentration of 4.8 nM in 2 mM citrate solution.

Unless otherwise noted, all solutions were prepared in Millipore water (18.2 MΩ·cm at 25°C) and all samples were prepared in a buffer consisting of 5 mM citrate—5 mM NaCl buffer at pH 6.5 (herein, citrate buffer). The pH of the buffer was adjusted through dropwise addition of 0.1 M and 1.0 M NaOH. Stock solutions of HSA and gHSA were prepared to a concentration of 5 μM in Millipore water. Stocks were aliquoted and frozen at −20°C for later use.

Human hepatoma (HepG2) cells were obtained from ATCC (Manassas, VA); Dulbecco’s modified Eagle medium (DMEM) from Mediatech (Manassas, VA); fetal bovine serum and penicillin—streptomycin from Invitrogen (Carlsbad, CA); sodium pyruvate and MEM non-essential amino acids from Life Technologies (Carlsbad, CA); trypsin/EDTA from CellGro (Manassas, VA); and WST cell proliferation assay kit from Dojindo Molecular Technologies (Rockville, MD).

### 2.2 Preparation of AgNPs-HSA and AgNPs-gHSA

The various techniques used across this study have different sample concentration requirements, so the concentration of AgNPs, HSA, and gHSA were varied to improve the signal-to-noise for each experiment. Specifically, for dynamic light scattering (DLS), zeta potential, UV-vis spectroscopy, and dissolution studies the concentration ratio was maintained as approximately 7×10^4^:1 protein:AgNPs, which was achieved by incubating 24 pM. AgNPs with 1.5 μM HSA or gHSA. For cell viability studies the concentration ratio was also approximately 7 × 10^4^:1 protein:AgNPs, which was achieved by incubating 10 pM. AgNPs with 0.70 μM protein or by incubating 100 pM. AgNPs with 7.0 μM protein. Langmuir adsorption isotherms were performed using a concentration ratio of 2 × 10^4^:1 protein:AgNPs, which was achieved by incubating 24 pM. AgNPs with varying concentrations of protein (25–500 nM). The decrease in the protein:AgNP ratio was necessary to achieve optimal binding conditions for quantitative analysis. For CE, the AgNP concentration was increased to improve detection sensitivity while the protein concentration was decreased to maintain the separation efficiency, resulting in a lower molar ratio than used in the other experiments. CE studies were performed using a concentration ratio of 6 × 10^2^:1 protein:AgNPs, which was achieved by incubating 24 pM. AgNPs with varying concentrations of protein (50–150 nM). Finally, for circular dichroism (CD) studies, the protein concentration was increased to improve detection sensitivity while the AgNP concentration was decreased to minimize interference due to light scattering from the NPs, which also resulted in a lower molar ratio than used in the other experiments. CD studies were performed using a concentration ratio of 3 × 10^2^:1 protein:AgNPs, which was achieved by incubating 3.0 nM AgNPs with 0.94 μM protein.

### 2.3 AgNP characterization

AgNPs were characterized using dynamic light scattering (DLS) to measure the hydrodynamic diameter, polydispersity index (PDI) and zeta potential. Measurements were recorded with a Malvern Zetasizer Nano-ZS instrument (Malvern, PA). All samples were prepared with a citrate buffer that was twice filtered with a 0.2 μm nylon syringe filter. Triplicate samples of AgNPs were prepared to a final concentration of 24 pM and contained 0 or 1.5 μM HSA or gHSA. Samples were incubated in the dark at room temperature for 24 or 72 h prior to analysis. DLS measurements were recorded using 173° backscatter mode after temperature equilibration for 2 min at 25°C. For each replicate sample, 11 sub-runs were recorded per measurement and 5 measurements were recorded and averaged. Reported values represent the average and standard deviation of three preparative replicates of AgNPs, AgNPs-HSA, and AgNPs-gHSA. Zeta potential measurements were recorded in the same way, but the number of sub-runs per measurement was set to automatic and constrained between 10–100. A Pd dip cell was used to measure zeta potential and values were determined using the Smoluchowski equation.

### 2.4 UV-vis spectroscopy

UV-vis experiments were performed using a Cary UV-vis spectrophotometer (Agilent Technologies, Inc.). AgNPs were prepared to a final concentration of 24 pM. in citrate buffer and were titrated with HSA or gHSA in the concentration range 0–500 nM. All samples were prepared in triplicate and incubated for 24 h in the dark at room temperature. All samples were analyzed in a semi-micro quartz cuvette with a 1 cm pathlength. Absorbance spectra were recorded from 400—450 nm at 60 nm·min^−1^. The shift in the localized surface plasmon resonance (LSPR) band was used to determine the association constant, *K*_a_, for the formation of the AgNP-protein complex according to (Boulos et al., 2013; Dennison et al., 2017; Boehmler et al., 2020):

$$\frac{∆\lambda }{{∆\lambda }_{\max}}=\frac{K_{a}C_{p}}{1+K_{a}C_{p}}$$ (1)

where Δ*λ* is the shift in the LSPR band relative to the sample without protein, Δ*λ*_max_ is the maximum shift in the LSPR band, and *C*_p_ is the protein concentration. Qualitative absorbance spectra of AgNPs were also recorded using the same concentration ratio of protein:AgNPs used in other studies. Specifically, 24 pM. AgNPs were incubated for 24 h in the dark at room temperature in buffer alone or in the presence of 1.5 μM HSA or gHSA. Absorbance spectra were recorded from 350 to 500 nm using a scan rate of 300 nm·min^−1^.

### 2.5 Capillary electrophoresis (CE)

CE was carried out on a P/ACE MDQ Plus capillary electrophoresis system from AB SCIEX. An uncoated fused silica capillary was used with an inner diameter of 50 μm. The total length of the capillary was 60.2 cm and the length to the detector was 50.0 cm. The separation buffer consisted of 5 mM Tris—500 mM glycine with a pH of 7.8. Each day the capillary was flushed successively for 10 min with Millipore water, 10 min with 0.1 M NaOH, 10 min with Millipore water, and 20 min with separation buffer. Between each run, the capillary was flushed for 1 min with Millipore water and 2 min with separation buffer. The separation buffer and the solutions used for rinsing the capillary were filtered with a 0.2 μm nylon syringe filter prior to use. Triplicate samples of AgNPs were prepared to a final concentration of 240 pM. in the separation buffer and contained 0, 50, 100, or 150 nM HSA or gHSA. All samples were incubated in the dark at room temperature for at least 30 min prior to analysis. Samples were injected hydrodynamically at 4 psi for 5 s (≈35 nL injection volume) and a 25 kV separation voltage was applied (applied field ≈ 415 V/cm). Each replicate sample was subjected to triplicate CE analysis to obtain reliable parameters for quantitative analysis, as described below.

Non-equilibrium capillary electrophoresis of equilibrium mixtures (NECEEM) was used to characterize AgNP-protein complex formation (Berezovski and Krylov, 2002; Berezovski et al., 2003; Krylov and Berezovski, 2003; Okhonin et al., 2004; Riley et al., 2018). In accordance with NECEEM theory, electropherograms were integrated to determine the areas under the unbound AgNP peak (*A*_1_), the AgNP-protein complex peak (*A*_2_), and in the region of dissociation (*A*_3_; Supplementary Figure S3). These peak areas were used to calculate the ratio of unbound to bound AgNPs, according to:

$$R=\frac{A_{1}}{A_{2}+A_{3}}$$ (2)

Then, using the calculated value of *R* and the initial concentrations of protein and AgNPs, [P]_0_ and [AgNPs]_0_, respectively, the dissociation constant was calculated, according to:

$$K_{d}=\frac{{\left[P\right]}_{0}\left(1+R\right)-{\left[AgNPs\right]}_{0}}{1+\frac{1}{R}}$$ (3)

To determine the rate constant for AgNP-protein complex dissociation, *k*_off_, CE electropherograms were fit in region *A*_3_ according to:

$$I_{t}=I_{t_{0}}e^{\left(k_{off}\left(\frac{t_{AgNP-P}}{t_{AgNP}-t_{AgNP-P}}\right)\left(t-t_{0}\right)\right)}$$ (4)

where *I*_t_ is the absorbance intensity at some point in time *t* and *I*_t0_ is the intensity of the absorbance signal at time *t*_0_. Time *t*_0_ represents the beginning of the exponential decay between the unbound AgNP peak at time *t*_AgNP_ and the AgNP-protein complex at time *t*_AgNP-P_ (Supplementary Figure S3). Using the calculated values of *K*_d_ and *k*_off_, the rate constant for AgNP-protein complex association, *k*_on_, was calculated according to:

$$k_{on}=\frac{k_{off}}{K_{d}}$$ (5)

### 2.6 Circular dichroism (CD) spectroscopy

Samples to be analyzed by CD were prepared by diluting HSA or gHSA to a concentration of 0.94 μM in 20 mM sodium phosphate buffer at pH 7.4. Samples were analyzed in a cylindrical quartz cuvette with a 1 mm pathlength and analyzed with an Olis Rapid-Scanning monochromator. A subsequent scan was taken after the addition of AgNPs to a final concentration of 3.0 nM and for a total sample volume of 280 μL. Measurements were recorded in the wavelength range 185–260 nm and the number of increments was set to 150. Consistent with previous studies of this kind (Fleischer and Payne, 2014), spectra were acquired in millidegrees (θ_obs_) and converted to mean residue ellipticity ( $\left[\theta \right]$ ), according to:

$$\left[\theta \right]=\frac{{MW\;\left[\theta \right]}_{obs}}{10\;l\;C\;n}$$ (6)

The mean residue ellipticity (units of degrees cm^2^ dmol^-1^) is a function of the observed signal in millidegrees, the average molecular weight of the protein (MW), path length (*l* in cm), protein concentration (*C* in g/L), and the total number of amino acids (*n*). The % α-helicity of the protein was determined according to (Adler et al., 1973):

$$\%\;\alpha \;helicity=\frac{-{\left[\theta \right]}_{MRE}-4000}{3300-4000}$$ (7)

The percent α-helicity of a protein is a function of the mean residue ellipticity at 208 nm ([θ_MRE_]) minus the contribution from the β-form and random coil conformations at 208 nm (4000). The observed value is compared to the mean residue ellipticity of a pure α-helix protein (33000).

### 2.7 Cell culture and WST assay

HepG2 cells were maintained and grown in 100 mm tissue culture dishes (Greiner, Bio-One, Monroe, CA, United States) using DMEM supplemented with 10% fetal bovine serum, sodium pyruvate, MEM non-essential amino acids, and 1% penicillin-streptomycin, at 37°C in a 5% CO_2_ humidified environment. The cells were grown to 70%–80% confluence (in approximately 7–10 days) and passaged using 0.25% trypsin/EDTA. For cell viability assays, cells (15,000 cells per well) were seeded in 96-well flat bottom plates and allowed to proliferate for 48 h. Cells were then washed with culture media alone (no AgNP or protein control), with culture media containing 0.70 or 7.0 μM HSA or gHSA (no AgNP control), or with culture media containing 10 pM or 100 pM of uncoated or protein-coated AgNPs (200 μL total volume per well). All samples were prepared in triplicate. Protein-coated AgNPs for the cell viability assays were prepared by reacting 100 pM AgNPs with 7.0 μM HSA or gHSA or 10 pM AgNPs with 0.7 μM HSA or gHSA for 10 min in DMEM. Control cells and cells exposed to the AgNP preparations were incubated for either 24 h or 72 h at 37°C in a humidified atmosphere containing 5% CO_2_ *.* At collection, the cells were washed with media to remove the NPs and eliminate any interference due to the NPs.

Cell viability was then measured using the commercially available WST assay according to the manufacturer’s instructions. WST solution (20 μL) was added to cells at a 1:10 dilution in DMEM (200 μL total volume per well), followed by a 2-h incubation period at 37°C in a humidified atmosphere containing 5% CO_2_. Absorbance was measured at 570 nm using a Tecan Infinite 200 PRO plate reader (Tecan, Switzerland). Background absorbance due to NPs was recorded using no cell controls for all the NP preparations and subtracted from the absorbance values of the experimental samples. Cell viabilities (percent) were thereafter calculated relative to controls not treated with AgNPs or proteins.

### 2.8 Linear sweep stripping voltammetry (LSSV)

LSSV was carried out as previously reported (Hui et al., 2019; Boehmler et al., 2020). Briefly, a three-electrode setup consisting of a glassy carbon working electrode, Ag/AgCl reference electrode, and Pt wire counter electrode was used to carry out the analysis. Measurements were recorded using a BASi Epislon Eclipse potentiostat and C-3 Cell Stand from BioAnalytical Systems, Inc., (West Lafayette, IN). All solutions were sparged with N_2_ (*g*) for 10 min and had a final dissolved oxygen concentration of approximate 8.0 mg·L^−1^. Each day, the working electrode was polished with 0.05 μm alumina polish and conditioned using 200 cycles of cyclic voltammetry from −0.5 to 0.35 V at 0.3 V·s^−1^. Between experiments, all electrodes were rinsed thoroughly with Millipore water, while the working electrode was placed in a 35% nitric acid solution for 30 s, rinsed thoroughly with Millipore water, and sonicated in Millipore water for 30 s. Stripping voltammetry was carried out by deposition at −0.5 V for 30 s with stirring, followed by a 5 s quiet time, and a linear sweep from −0.5 to 0.35 V at 0.1 V·s^−1^. A same-day matrix-matched calibration curve was prepared with Ag(I) standards in the concentration range 0–240 μg·L^−1^.

The initial dissolution rate of AgNPs, AgNPs-HSA, and AgNPs-gHSA was measured by placing citrate buffer and the appropriate volume of protein (final concentration of 300 nM) into the electrochemical cell and sparging for 10 min. Then, the appropriate volume of AgNPs was added so that the final concentration was 4.7 pM (protein:AgNP ratio of ≈7 × 10^4^:1) and LSSV was carried out every 5 min for 2 h. The experiment was repeated in triplicate and the resulting dissolution curves (plots of [Ag(I)]_dissolved_ vs. time) were used to determine the dissolution rate constant, *k*_dissolution_, according to:

$$\ln\left(1-\frac{{\left[Ag\left(I\right)\right]}_{t}}{{\left[AgNP\right]}_{0}}\right)=-k_{dissolution}t$$ (8)

The percentage of dissolved silver, %AgNPs_dissolved_, was determined by normalizing the [Ag(I)]_dissolved_ measured at the end of the dissolution kinetics experiment (*t* = 2 h) to the initial silver concentration placed into solution [Ag]_0_, according to:

$${\%\;AgNPs}_{dissolved}=\frac{{\left[Ag\left(I\right)\right]}_{dissolved,t=2h}}{{\left[Ag\right]}_{0}}\times 100$$ (9)

The dissolution of AgNPs was also monitored after 24 and 72 h incubation in buffer alone or buffer containing protein. Six samples of AgNPs, AgNPs-HSA, and AgNPs-gHSA were prepared in citrate buffer with final concentrations of 24 pM. AgNPs and 1.5 μM protein (protein:AgNP ratio of ≈7 × 10^4^:1) and incubated in the dark at room temperature. After 24 h, three replicates of each sample were analyzed using LSSV to determine the percentage of dissolved Ag(I) using same-day matrix matched calibration curves. After 72 h from the initial exposure, the remaining three replicates of each sample were analyzed in the same manner.

## 3 Results

### 3.1 Characterization of AgNP-protein complex formation

**FIGURE 1:**
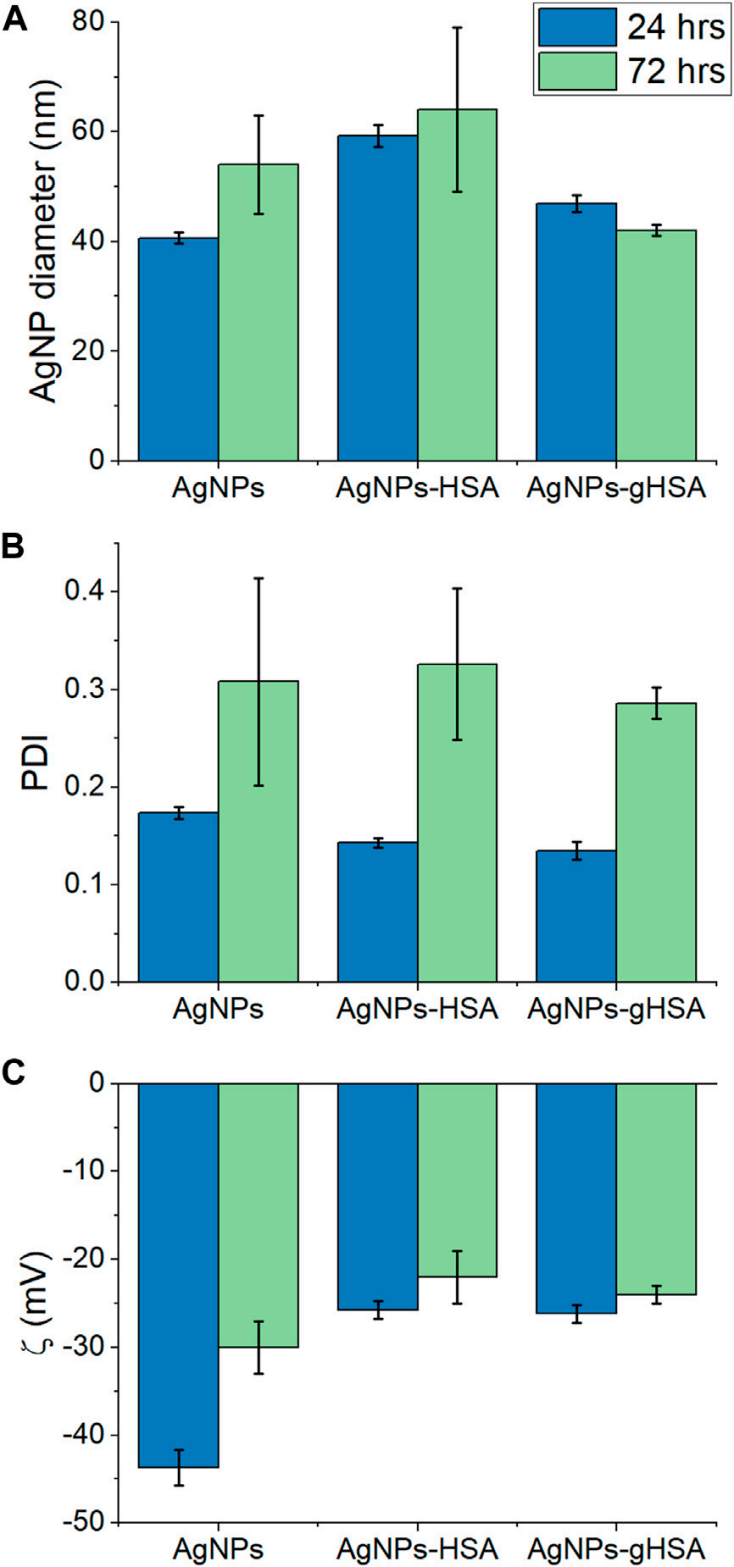
**(A)** Hydrodynamic diameter **(B)** PDI, and **(C)** zeta potential of AgNPs after 24 and 72 h incubation in buffer alone or buffer containing HSA or gHSA. AgNPs were prepared to a final concentration of 24 pM and the proteins to a final concentration of 1.5 μM. All solutions were prepared in 5 mM citrate—5 mM NaCl buffer (pH 6.5).

The formation of AgNP-protein complexes was confirmed qualitatively by measuring the hydrodynamic diameter, PDI, and zeta potential of AgNPs alone, in the presence of HSA, or in the presence of gHSA. Samples were analyzed at time points of 24 and 72 h following incubation to assess the stability of the AgNPs over time. After 24 h, an increase in the hydrodynamic diameter of AgNPs was observed in the presence of both HSA and gHSA relative to the no protein control (Figure 1A; Supplementary Figure S1; Supplementary Table S1). However, the magnitude of this increase differed between the two proteins, with the addition of HSA resulting in a larger increase in AgNP diameter (≈20 nm) and the addition of gHSA resulting in a more modest increase (≈7 nm). Differences in the thickness of the HSA and gHSA PCs formed on the AgNP surface could be a result of differences in the protein structure and/or orientation on the surface (*vide infra*). After 72 h, an increase in the diameter of AgNPs was observed suggesting slight aggregation of the particles over time. No significant differences were noted for the protein-coated AgNPs after 72 h, suggesting that the coronas was relatively stable and prevented significant aggregation of the AgNPs (Figure 1A; Supplementary Figure S1; Supplementary Table S1).

After 24 h incubation, the increase in AgNP diameter upon addition of HSA or gHSA was accompanied by either no change or a slight decrease in the PDI, providing further evidence that the formation of the AgNP-PCs provided some steric stabilization of the colloidal suspension (Figure 1B; Supplementary Table S1). Based on the decrease in PDI values, the degree of steric stabilization conferred by the proteins was more significant for gHSA than for HSA (Supplementary Table S1). After 72 h, the PDI value for all AgNPs increased, suggesting changes in the homogeneity of the samples over time and the potential for particle-particle or protein-protein aggregation. The formation of the AgNP-protein complex was further demonstrated by a decrease in the AgNP zeta potential (toward more positive potential) after 24 h incubation with HSA or gHSA (Figure 1C; Supplementary Table S1). After 72 h incubation, the zeta potential significantly decreased for the sample of AgNPs alone, further supporting the likelihood that the uncoated AgNPs had begun to aggregate. The zeta potentials for the protein-coated AgNPs showed only a subtle decrease in magnitude, further supporting the stability conferred by the protein coating. Overall, only subtle differences were observed between the AgNP-HSA and AgNP-gHSA coronas and more significant changes were observed between uncoated and protein-coated AgNPs.

**FIGURE 2:**
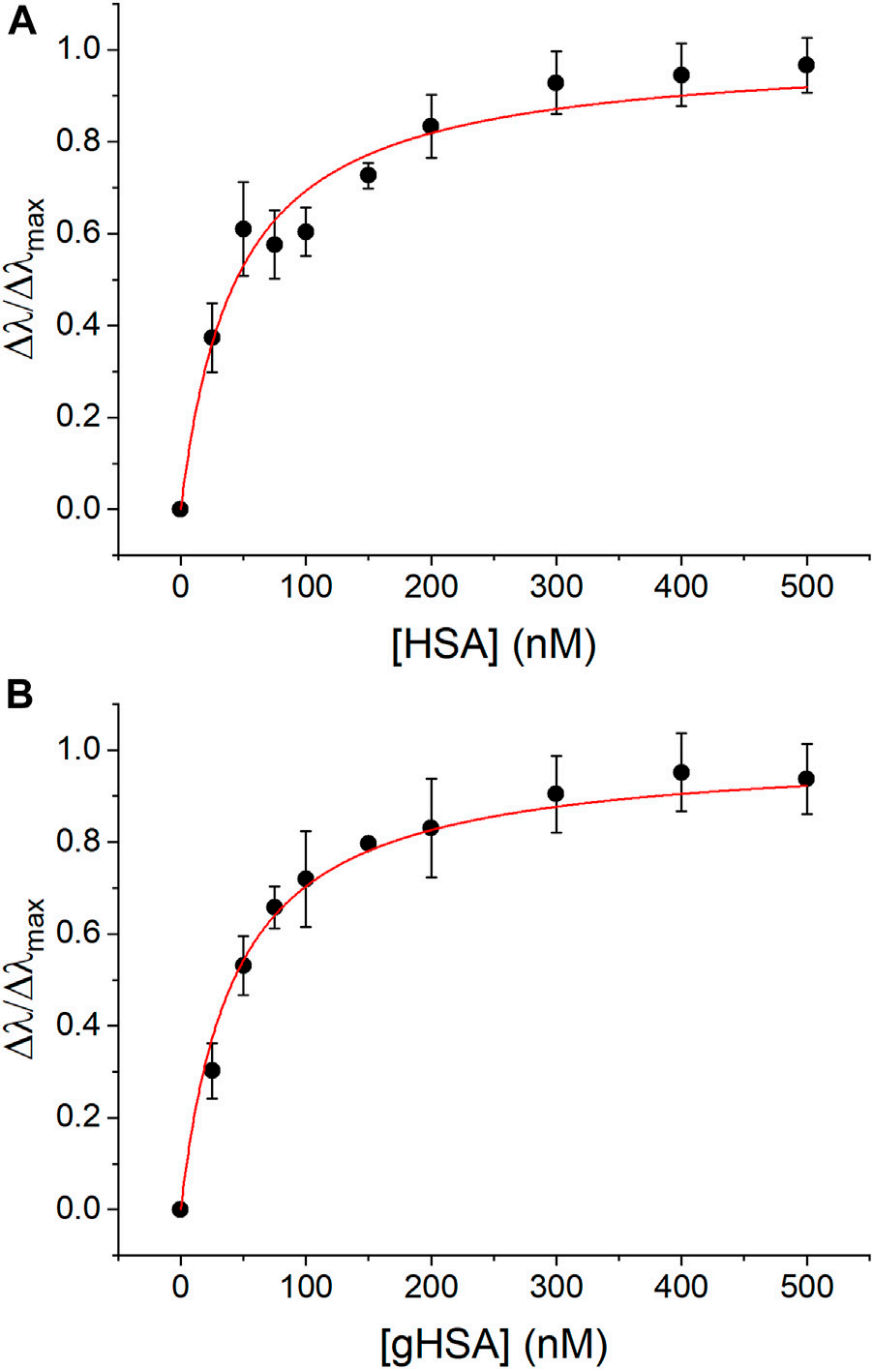
UV-vis Langmuir adsorption isotherms for complex formation between AgNPs and **(A)** HSA and **(B)** gHSA. The experimental data was fit using Eq. 1 (*R* ^2^ > 0.97). AgNPs were prepared to a concentration of 24 pM and the protein concentrations were as indicated. All solutions were prepared in 5 mM citrate—5 mM NaCl buffer (pH 6.5).

The formation of AgNP-protein complexes was also confirmed quantitatively using UV-vis spectroscopy and CE to measure association and rate constants for complex formation. The 40 nm AgNPs exhibited a prominent LSPR band at approximately 420 nm that shifted to longer wavelength in the presence of HSA and gHSA (Supplementary Figure S2). Langmuir adsorption isotherms were constructed to determine the association constant, *K*_*a*_, for AgNP-protein complex formation (Figure 2). The *K*_a_ values for HSA and gHSA were 2.3 (±0.2) × 10^7^ M^−1^ and 2.4 (±0.1) × 10^7^ M^−1^ (*n* = 3, *R* ^2^ > 0.97), respectively, suggesting that both proteins had similar affinity for the AgNP surface (Table 1).

**TABLE 1:** Characterization of AgNP-HSA and AgNP-gHSA complex formation.

Protein	*K*_a_ (×10^7^ M^−1^)		*k*_off_ ^b^ (s^−1^)	*k*_on_ ^b^ (x10^5^ M^−1^·s^−1^)
	UV-vis^a^	CE^b^		
HSA	2.3 ± 0.2	2.5 ± 1.0	0.041 ± 0.008	1.1 ± 0.5
gHSA	2.4 ± 0.1	2.2 ± 1.1	0.062 ± 0.024	1.3 ± 0.8

^a^ Experimental conditions were as reported in Figure 2 and values were calculated using Eq. 1. Values are the average and standard deviation of 3 trials.

^b^ Experimental conditions were as reported in Figure 3 and values were calculated using Eqs 2–5. Values are the average and standard deviation of 9 trials (three replicate experiments performed at each of three protein concentrations).

The formation of AgNP-protein complexes was further quantified using CE and NECEEM analysis. While NECEEM theory was initially developed to measure DNA-protein complexes, it has recently been demonstrated as an effective tool to measure the NP-PC (Riley et al., 2018). According to NECEEM theory, injection of the equilibrium mixture (containing unbound AgNPs, unbound protein, and AgNP-protein complexes) onto the capillary and application of the separation voltage will lead to a non-equilibrium condition, whereby each component of the mixture will migrate according to their unique electrophoretic mobilities. As a result, when the AgNP-protein complexes dissociate and the unbound AgNPs and protein migrate away from one another in the capillary, they are no longer proximate and cannot reestablish the AgNP-protein complex (i.e., a non-equilibrium condition) (Berezovski and Krylov, 2002; Berezovski et al., 2003; Krylov and Berezovski, 2003; Okhonin et al., 2004; Riley et al., 2018). By directly monitoring the dissociation of AgNP-protein complexes, both the dissociation constant (*K*_d_) and the on/off rate constants (*k*_on_/*k*_off_) can be determined in a single experimental run (Berezovski and Krylov, 2002).

**FIGURE 3:**
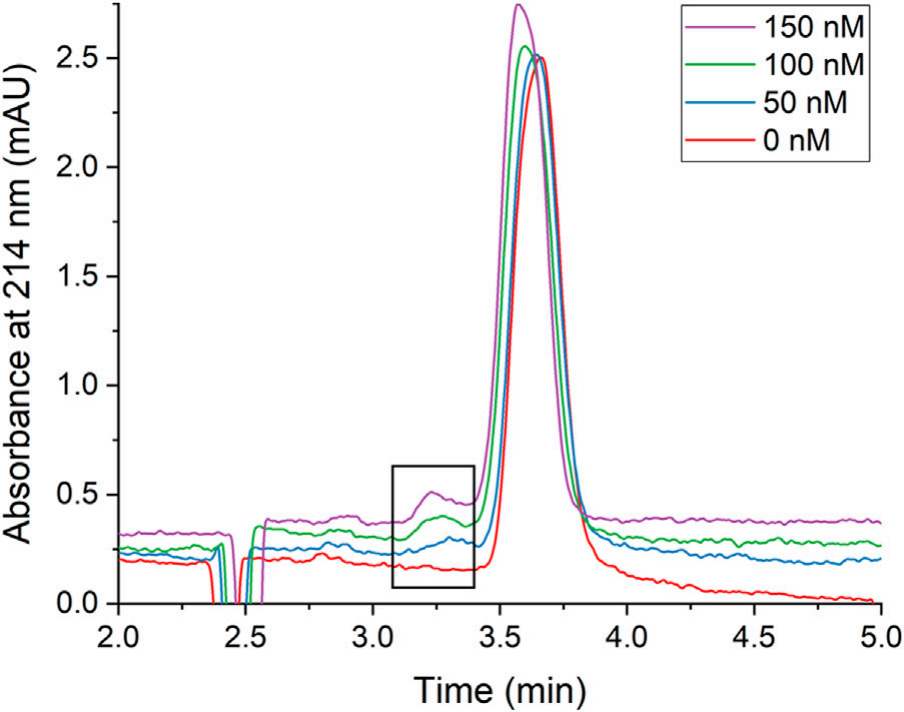
Representative CE electropherograms of AgNPs in the presence of increasing concentrations of HSA. The boxed region highlights the AgNP-HSA complex peak which increases with increasing [HSA]. Electropherograms are vertically offset for clarity. AgNPs were prepared to a concentration of 240 pM and the protein concentrations were as indicated. All solutions were prepared in 5 mM Tris—500 mM Gly buffer (pH 7.8).

In this work, only the AgNPs generated an absorbance signal in the electropherogram because the concentration and volume of protein injected onto the capillary was too low to be detected. In the absence of protein, a single peak at approximately 3.7 min was observed and attributed to the AgNPs (Figure 3, red trace). With the addition of HSA, a new peak was observed at approximately 3.3 min and was attributed to the AgNP-HSA complex due to an observed increase in absorbance intensity of the peak with increasing protein concentration (Figure 3). Electropherograms for NECEEM analysis of AgNPs-gHSA complexes exhibited similar features (Supplementary Figure S4). By applying quantitative NECEEM analysis (Eqs 2, 3; Supplementary Figure S3), the *K*_*d*_ values of AgNP-HSA and AgNP-gHSA complexes were obtained and then converted to *K*_a_ values for ease of comparison. The values obtained by NECEEM were consistent with those obtained using UV-vis spectroscopy within error, confirming that HSA and gHSA have similar affinity for the AgNP surface (Table 1). NECEEM analysis of the dissociation kinetics was also carried out (Eqs 4, 5; Supplementary Figure S3), and likewise, no significant differences were noted between the *k*_*on*_ and *k*_*off*_ values determined for the AgNP-HSA and AgNP-gHSA complexes. Generally, the values obtained suggest fast adsorption and slow desorption of the proteins from the AgNP surface (Table 1).

**FIGURE 4:**
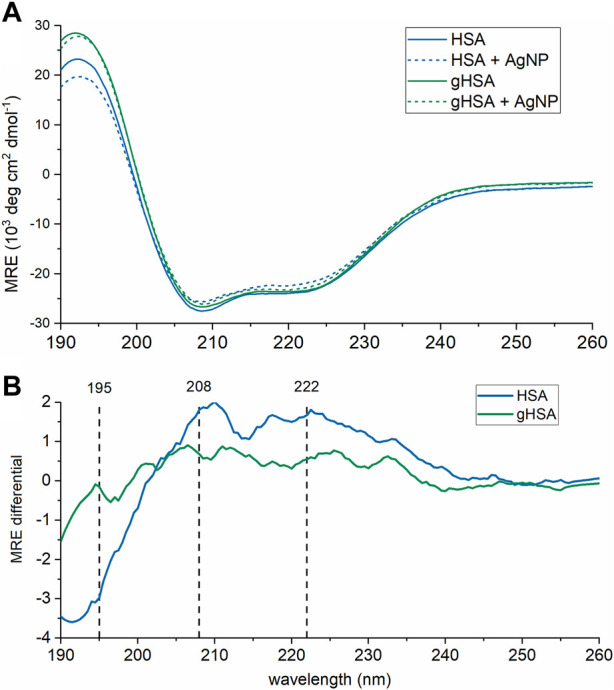
CD spectra of HSA and gHSA (blue and green, respectively) alone (solid lines) and with AgNPs (dashed). Spectra, in units of mean residue ellipticity (MRE), are the average of 3 consecutive scans. **(A)** Raw CD spectra. **(B)** CD difference spectra calculated by subtracting the spectrum of HSA (blue) or gHSA (green) from that in the presence of AgNPs. The black vertical lines correspond to the spectral peaks at 195, 208, and 222 nm. The protein concentration was 0.94 μM and the AgNP concentration was 3.0 nM. All solutions were prepared in 5 mM sodium phosphate buffer (pH 7.4).

Finally, changes to protein structure upon PC formation were probed using CD spectroscopy (Figure 4A; Table 2). A comparison of the two proteins in the absence of AgNPs revealed slight differences in the protein secondary structure, with gHSA exhibiting slightly greater *α*-helicity than HSA (Figure 4A; Table 2). Others have noted changes in HSA structure upon glycation, where the degree and exact nature of those changes depend upon the type of monosaccharide, concentration, and length of exposure (Nakajou et al., 2003; Qiu et al., 2021). Upon the addition of AgNPs to each protein solution, the *α*-helical character of the protein decreased, as measured by changes in the ellipticity at 208 and 220 nm and the calculated % α-helicity (Figure 4B; Table 2). While CD suffers from low sensitivity and an inability to distinguish free from surface-adsorbed protein, it can provide qualitative and semi-quantitative analysis of changes in protein secondary structure, which may be attributed to the formation of NP-protein complexes. Generally, changes in protein ellipticity can be taken as further evidence of the formation of the NP-PC. Our results demonstrate that the reduction of *α*-helicity upon interaction with AgNPs was slightly larger for HSA than for gHSA, which suggests possible differences in the surface interaction of HSA and gHSA due to protein glycation.

**TABLE 2:** Percent α-helicity for HSA and gHSA alone and in the presence of AgNPs^a^.

Sample	% α-helicity	
	Alone	With AgNPs
HSA	53	46
gHSA	67	64

^a^ Percent α-helicity was calculated using Eqs 6, 7.

### 3.2 Cell viability studies

Studying the effect of protein coating on cell viability is important because NPs used in the body will inevitably be coated with a PC when they come in contact with biological fluids (e.g., blood) or culture medium. Such a protein coating has been shown to influence targeting abilities, cellular uptake, and immunotoxicity of NPs (Monteiro-Riviere et al., 2013; Nierenberg et al., 2018). Thus, we hypothesized that the HSA and gHSA PCs would each alter cell toxicity, but that there would be no significant differences between their impact since biophysical analyses suggested that the coronas had remarkably similar properties under the chosen experimental conditions. To evaluate this hypothesis, we investigated the role of protein coatings on the toxicity of AgNPs to human cells. We chose liver hepatocellular carcinoma (HepG2) cells as our cell model, as they are widely used in the literature to determine toxicity. The liver has also shown to be a target organ for several NPs, making liver cells biologically relevant for toxicity studies (Balasubramanian et al., 2010; Lankveld et al., 2010; Khosravi-Katuli et al., 2017; Wu and Tang, 2018).

**FIGURE 5:**
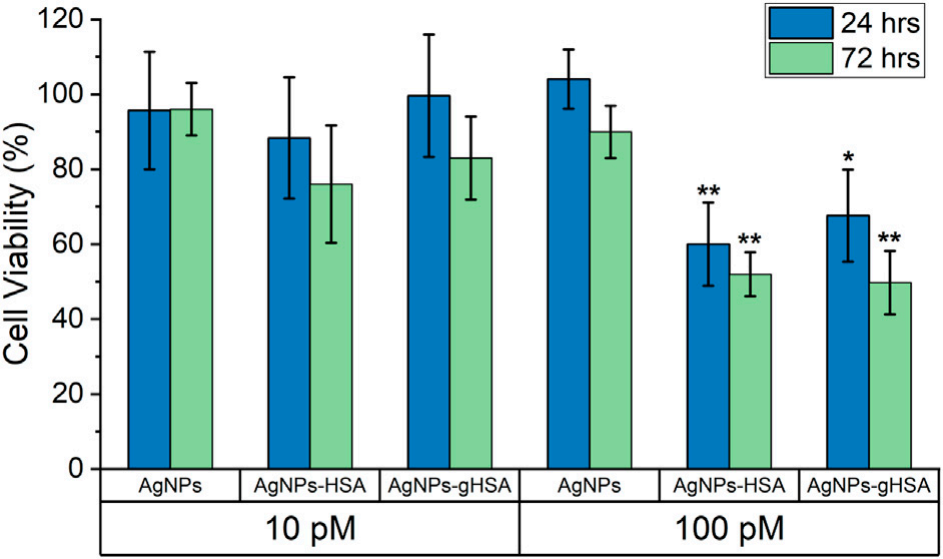
Percent cell viability of HepG2 cells, as determined by WST assay, after 24 and 72 h exposure to 10 pM and 100 pM AgNPs, AgNPs-HSA, or AgNPs-gHSA. Cell viability data was corrected by subtracting background absorbance due to AgNPs and normalized against HepG2 cells incubated in culture media alone (no protein and no AgNPs). Error bars represent the standard deviation of three samples and statistical significance was independently evaluated for each protein condition relative to the no protein control using a two-tailed *t*-test evaluated at the 95% (*) or 99% (**) confidence interval.

HepG2 cells were incubated with varying concentrations of AgNPs under three conditions: AgNPs exposed to HSA, gHSA, and no protein (uncoated AgNPs). Control studies included incubation of HepG2 cells in culture media alone or with culture media containing different concentrations of HSA or gHSA. After 24 or 72 h incubation, a WST assay was performed to evaluate cell viability. As expected, relative to cells incubated in cell culture media alone, no toxicity was observed for cells exposed to just HSA or gHSA with no AgNPs (Supplementary Table S2). The toxicity due to the addition of AgNPs was concentration dependent with higher levels of toxicity observed at higher AgNP concentrations. Interestingly, the results revealed increased toxicity for both HSA-coated and gHSA-coated AgNPs relative to uncoated AgNPs (Figure 5; Supplementary Table S3).

### 3.3 Towards a mechanistic understanding of protein mediated AgNP toxicity

As a first step towards investigating the mechanism by which the HSA and gHSA PCs decrease HepG2 cell viability, LSSV was used to measure AgNP dissolution in the absence and presence of both proteins. For all LSSV analyses, same-day matrix-matched calibration curves were obtained, which was important to account for any impact of the proteins on the electrochemical signal. All calibration curves had sub-micromolar LODs (typically around 0.10–0.30 μM) under all experimental conditions evaluated (Supplementary Tables S4, S5) suggesting that the protein did not unduly effect LSSV analyses.

First, the initial rate of dissolution was measured for AgNPs alone or in the presence of HSA or gHSA over the first 2 h following incubation. The dissolution rate constant, *k*_dissolution_, of AgNPs alone was 1.6 × 10^−4 ^min^−1^ indicating rapid dissolution of AgNPs after dilution in buffer (Supplementary Figure S5; Supplementary Table S4). In contrast, AgNPs added to buffer containing either HSA or gHSA exhibited slow dissolution and a nearly constant concentration of Ag(I) over the 2 h analysis period, so dissolution rate constants were unable to be measured (Supplementary Figure S5; Supplementary Table S4). At the conclusion of the 2 h AgNP dissolution experiment, the faster dissolution kinetics of AgNPs in the no protein condition led to a 2-fold increase in the percentage of dissolved Ag(I) relative to AgNPs in the presence of HSA and gHSA (Supplementary Figure S5; Supplementary Table S4).

**FIGURE 6:**
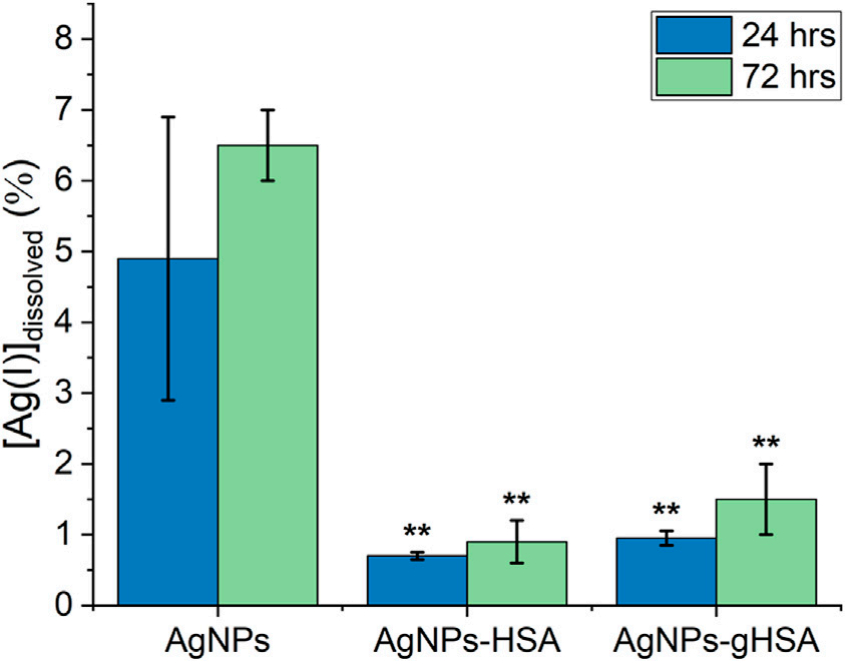
Percent dissolution of AgNPs after 24 and 72 h incubation in buffer alone or buffer containing HSA or gHSA. Dissolution was measured by LSSV and the percentage of dissolved Ag(I) was quantified using Eq. 9. AgNPs were prepared to a final concentration of 24 pM and the proteins to a final concentration of 1.5 μM. All solutions were prepared in 5 mM citrate—5 mM NaCl buffer (pH 6.5). Statistical significance was independently evaluated for each protein condition relative to the no protein control using a two-tailed *t*-test evaluated at the 99% (**) confidence interval.

Analysis of AgNP dissolution behaviors was extended by measuring the percentage of dissolved Ag(I) after 24 and 72 h incubation in buffer alone, or in buffer containing HSA and gHSA. These time points were chosen to exactly mimic the conditions used for the cell viability assay. Under all experimental conditions a slight increase in the percentage of dissolved Ag(I) was observed after 72 h incubation relative to 24 h (Figure 6; Supplementary Table S5). This increase was most significant for the AgNPs incubated in buffer only, consistent with kinetic dissolution experiments that showed the AgNPs alone undergo much faster dissolution. Relative to the AgNPs alone, AgNPs incubated with HSA or gHSA exhibited a statistically significant decrease in the percentage of dissolved Ag(I) at both 24 and 72 h following incubation (Figure 6; Supplementary Table S5). Specifically, the dissolution of AgNPs in the presence of protein was ≈5-fold lower than the no protein control after 24 h and ≈6-fold lower after 72 h.

## 4 Discussion

Biophysical analyses demonstrated subtle differences in the HSA and gHSA PCs, including the thickness of the corona measured using DLS (Figure 1A) and changes in the protein secondary structure upon interaction with AgNPs measured using CD spectroscopy (Figure 4). Inherent differences in the chemical composition and secondary structure of HSA and gHSA due to glycation may alter the orientation of the proteins on the AgNP surface, which may contribute to the observed differences in the corona thickness in DLS analyses. In a previous study, the thickness of the PC formed on quantum dots varied between unmodified HSA and HSA modified either through succinylation or amination due to differences in protein orientation on the surface (Treuel et al., 2014). Further, the subtle increase in α-helicity upon glycation of HSA measured using CD spectroscopy has been previously observed and is presumed to be due to slight structural changes conferred by the glycation of specific amino acid residues (Coussons et al., 1997; Barzegar et al., 2007). Consistent with previous results, a decrease in *α*-helicity of HSA and gHSA upon interaction with AgNPs suggests slight unfolding of the protein as it adsorbs to the NP surface, presumably due to favorable interactions (e.g., hydrophobic) between specific regions of the protein and the NP surface (Shang et al., 2007; Bardhan et al., 2009; Pan et al., 2012; Fleischer and Payne, 2014). For example, lysine residues are commonly glycated in HSA (Anguizola et al., 2013), which can lead to changes in protein structure that cause further changes in NP-protein interactions. Our results demonstrate that the reduction of *α*-helicity upon interaction with AgNPs was slightly larger for HSA than for gHSA, which is consistent with previous studies of transferrin interactions with citrate-coated AgNPs (Barbir et al., 2021), and further suggests possible differences in the surface interaction of HSA and gHSA due to protein glycation.

Quantitative UV-vis and CE studies (Figures 2, 3) showed no distinguishable differences between the equilibrium and rate constants for the formation of AgNP-HSA and AgNP-gHSA complexes. AgNP-protein association constants (*K*_a_ values) obtained in this work are also similar to those reported in the literature for BSA and 40 nm citrate-stabilized AgNPs (Dasgupta et al., 2016; Boehmler et al., 2020). Ultimately, under our experimental conditions, biophysical characterization indicates no significant differences in the formation of the AgNP-HSA and AgNP-gHSA complexes, but distinct changes in AgNP physical properties upon corona formation compared to protein-free conditions. Specifically, the formation of the HSA and gHSA PCs resulted in increased colloidal stability of the AgNPs as evidenced by less significant changes in AgNP diameter and zeta potential over time (Figures 1A, C). The stabilization of AgNPs due to the formation of the PC is consistent with a previous study that showed that the analogous protein bovine serum albumin (BSA) can stabilize AgNPs even in solution conditions that promote AgNP aggregation (Levak et al., 2017). Our overall observations are also consistent with a previous study conducted with SiO_2_ NPs, where only subtle changes in NP diameter, PDI, and zeta potential were observed between PCs with and without glycosylation, and more significant changes were observed between the NPs with and without protein (Wan et al., 2015).

The HSA and gHSA PCs also altered the reactivity of the AgNPs to HepG2 cells. Specifically, after 72 h exposure to protein-coated AgNPs, HepG2 cells showed an approximately 2-fold decrease in cell viability compared to uncoated AgNPs. These results indicate no observable differences in toxicity between AgNPs coated with HSA compared to gHSA, for the experimental conditions and endpoints evaluated herein, a conclusion consistent with the biophysical experiments. Notably, there are limited studies on the role of PC glycation in toxicity. For example, Wan et al. (2015) demonstrated increased cell membrane adhesion and uptake in macrophages upon removal of glycans from the human plasma PC on silica NPs. More recent studies with different NPs show that glycation can vary nanocarrier cellular uptake in different directions, depending on the protein (Ghazaryan et al., 2019). Given the differences across studies, including corona composition, NP types, and cell types, it is difficult to draw any meaningful connections. Further, both studies are limited to evaluation of the role of glycation in hard corona formation. Glycation of the PC may also influence soft corona formation and other toxicity related endpoints, which deserve further attention.

The observed increase in toxicity with a PC is consistent with a recent paper that shows an increase in toxicity for citrate-coated AgNPs when coated with serum proteins (Barbalinardo et al., 2018). This is not a consistently reported finding, as other studies have demonstrated that the PC may decrease cellular uptake of AgNPs and their toxicity (Monteiro-Riviere et al., 2013; Shannahan et al., 2015). The observed differences in toxic responses may be due to myriad of factors, including nanoparticle size and concentration, exposure times, different cell types, and choice of toxicity assays (Sohaebuddin et al., 2010; Kroll et al., 2011). Additional studies are necessary to further evaluate the source(s) of the differences, as well as the role of PC on the response of AgNPs to human cells. The oxidative dissolution of AgNPs to release bioactive Ag(I) is the primary contributor to AgNP antimicrobial properties. Ag(I) dissolved from AgNPs can interfere with transport proteins and enzymes in the respiratory chain reaction, compromise the proton-motive force, and interfere with phosphate uptake (Schreurs and Rosenberg, 1982; Dibrov et al., 2002; Holt and Bard, 2005; Lok et al., 2006). However, there is strong evidence that AgNPs also contribute to toxicity (Navarro et al., 2008; Li et al., 2017), which can proceed through direct disruption of the cell membrane and/or the generation of reactive oxygen species (ROS), which disrupt protein structures and interfere with DNA replication (Sondi and Salopek-Sondi, 2004; Choi et al., 2008; Smetana et al., 2008; Monteiro-Riviere et al., 2013; Barbalinardo et al., 2018; Nierenberg et al., 2018). Thus, to elucidate whether the decreased cell viability observed for protein-coated AgNPs was due to protein-induced enhancement in the dissolved Ag(I) concentration we measured the dissolution of AgNPs in the absence and presence of HSA or gHSA. After 2 h incubation, the AgNP only control underwent 2-fold greater dissolution than the protein-coated AgNPs, which increased to a factor of 5-fold greater dissolution after 24 h and 6-fold greater dissolution after 72 h (Supplementary Figure S5; Figure 6). Other reports in the literature highlight the concentration-dependence of protein-mediated AgNP dissolution. At low protein concentrations, where the AgNP surface is unsaturated or there is only monolayer surface coverage, proteins can enhance dissolution through a nucleophilic mechanism (Martin et al., 2014; Liu et al., 2018; Boehmler et al., 2020). At higher protein concentrations (like the ones used in this study), where the AgNP surface is fully saturated and a stable corona is formed, the surface-bound proteins form a protective coating that inhibits dissolution (Martin et al., 2014; Tai et al., 2014; Shannahan et al., 2015; Liu et al., 2018). Taken together, the protein-coated AgNPs were more toxic to HepG2 cells compared to the no protein control, even as the dissolution of protein-coated AgNPs was decreased relative to the no protein control. These results suggest that a NP- and PC-specific mechanism rather than a dissolution mechanism is likely responsible for the increased toxicity measured for protein-coated AgNPs. In fact, since the completion of this work, a recent study in the literature showed the colocalization of 20 nm and 100 nm citrate-stabilized AgNPs and the mitochondria of HepG2 cells, leading to greater mitochondrial damage and apoptosis relative to exposure to Ag(I) alone (Wang et al., 2022). This supports our hypothesis that a NP-specific mechanism is responsible for the observed toxicity of AgNPs to HepG2 cells. The present study extends this work by demonstrating that the HSA and gHSA coronas potentiate AgNP toxicity.

Previously reported biophysical studies using model lipid membranes can provide insights to the mechanism of NP- and PC-specific toxicity of NPs to cells. Previous studies demonstrated that very small (<5 nm) AgNPs can be trapped within liposomes and increase the fluidity of model lipid bilayers (Park et al., 2005; Bothun, 2008). Similarly, another study showed that 40 nm AgNPs coated with HSA can lead to increased bilayer fluidity (Chen et al., 2012), while a separate study showed that the interaction of HSA-coated AgNPs with model membranes is strongly dependent on solution pH (Wang et al., 2016). Few studies have examined protein-mediated interactions between AgNPs and model membranes with the level of detail needed to elucidate a full mechanism. However, more sophisticated experimental techniques have been developed to investigate NP-membrane interactions with AuNPs and can serve as a model for future work with AgNPs. Using model lipid membranes, researchers showed that AuNPs caused membrane disruption through lipid extraction. This process was strongly influenced by the particle capping agents, with more positively charged particles leading to greater lipid extraction from the membrane (Olenick et al., 2018; Zhang et al., 2018). Another study demonstrated the complexity of nano-bio interactions, whereby the AuNP surface properties influenced the composition of the PC, and the corona, in turn, impacted the interactions of the AuNPs with model membranes (Melby et al., 2017). Further adding to this complexity is the possibility that AuNPs can form PCs through extraction of peripheral membrane proteins (Melby et al., 2018), and the likelihood that a combination of all of these processes (and those yet to be studied) contribute to toxicity mechanisms *in vivo*.

## 5 Conclusion

This study serves as a first step to interrogating the role of protein modification, like glycation, in mediating PC formation and AgNP toxicity. We have provided robust, qualitative and quantitative characterization of the AgNP-HSA and AgNP-gHSA PCs. We have further demonstrated that, under our experimental conditions, glycation of HSA does not significantly alter the formation of the AgNP-PC or its impact on AgNP dissolution and toxicity to HepG2 cells. Instead, we have shown that both HSA- and gHSA-coated AgNPs are more toxic to HepG2 cells than uncoated AgNPs, and that the mechanism of toxicity cannot be simply explained by AgNP dissolution. Indeed, we observe decreased AgNP dissolution upon formation of the HSA and gHSA coronas at the same time that we observe increased cell toxicity, suggesting a NP- and PC-induced mechanism of toxicity. While glycation of HSA did not appear to have a significant impact in our study, the incorporation of protein modifications into the study of the PC is ultimately an important first step toward understanding the complete AgNP biocorona that includes proteins, metabolites, and lipids (Chetwynd and Lynch, 2020). Our study further highlights the need for continued experimentation and development of biophysical and analytical methods to elucidate the mechanism of protein-mediated AgNP toxicity.

## Data Availability

The raw data supporting the conclusion of this article will be made available by the authors, without undue reservation.
