# Supplementary material for: Silver nanoparticle interactions with glycated and non-glycated human serum albumin mediate toxicity
Source: Front Toxicol. 2023 Feb 28;5:1081753. doi: 10.3389/ftox.2023.1081753 (PMC10011623; doi:10.3389/ftox.2023.1081753)
Supplement: Supplementary file 1 [file DataSheet1.PDF]

ELECTRONIC SUPPLEMENTARY INFORMATION:
Silver nanoparticle interactions with glycated and nonglycated human serum albumin mediate toxicity

Hee-Yon Park[1], Christopher Chung[1], Madeline K. Eiken[2], Karl V. Baumgartner[2], Kira M. Fahy[2], Kaitlyn Q. Leung[2], Evangelia Bouzos[3], Korin E. Wheeler[2], Prashanth Asuri[3], Kathryn R. Riley[1*]

[1]Department of Chemistry & Biochemistry, Swarthmore College, Swarthmore, PA 19081, USA
[2]Department of Chemistry & Biochemistry, Santa Clara University, Santa Clara, CA 95053, USA
[3]Department of Bioengineering, Santa Clara University, Santa Clara, CA 95053, USA

CONTENTS

Figure S1. DLS histograms of AgNPs in the presence of HSA and gHSA

Table S1. DLS and zeta potential characterization of AgNPs in the presence of HSA and gHSA

Figure S2. UV-vis spectra of AgNPs in the presence of HSA and gHSA

Figure S3. Representative CE electropherograms demonstrating the application of NECEEM theory to determine dissociation and rate constants for AgNP-protein complexes

Figure S4. Representative CE electropherograms of AgNPs in the presence of increasing concentrations of gHSA.

Table S2. Percent cell viability of HepG2 cells in the presence of HSA and gHSA (no AgNPs)

Table S3. Percent cell viability of HepG2 cells in the presence of HSA- and gHSA-coated AgNPs

Figure S5. Representative kinetics curves for the initial dissolution of AgNPs (0-2h)

Table S4. Characterization of initial AgNP dissolution rates in the presence of HSA and gHSA

Table S5. Characterization of AgNP dissolution following 24h or 72h incubation with HSA and gHSA

[*]Corresponding author: Tel: +1-610-690-3904, email: kriley1@swarthmore.edu

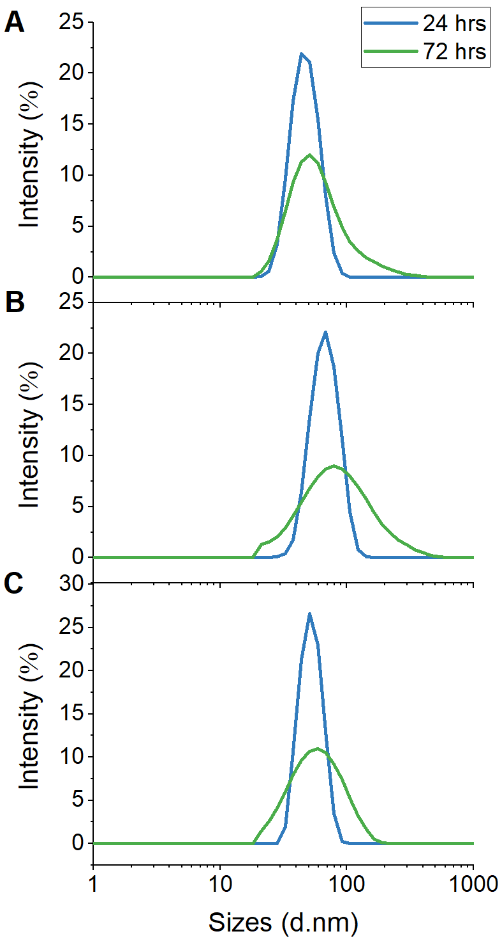

Figure S1. DLS histograms of **(A)** AgNPs alone (no protein), **(B)** AgNPs-HSA, and **(C)** AgNPs-gHSA 24 and 72h after exposure. AgNPs were prepared to a concentration of 24 pM and the protein concentrations were 1.5 μM. All solutions were prepared in 5 mM citrate – 5 mM NaCl buffer (pH 6.5) and incubated in the dark at room temperature. Histograms were generated by averaging measurements from three independently prepared replicate samples.

Table S1. Characterization of AgNPs 24 and 72h after exposure to HSA and gHSA[a]

sample	exposure time (h)	$d_{AgNP, DLS}$ (nm)	PDI	ζ(mV)
no protein	24	40.6 ± 0.3	0.174 ± 0.006	-44 ± 2
	72	54 ± 9	0.300 ± 0.100	-30 ± 3
HSA	24	59.2 ± 0.3	0.143 ± 0.005	-26 ± 1
	72	64 ± 15	0.330 ± 0.080	-22 ± 3
gHSA	24	46.9 ± 0.1	0.135 ± 0.009	-26 ± 1
	72	42 ± 1	0.290 ± 0.016	-24 ± 1

[a]Experimental conditions are as reported in Figures 1 and S1.

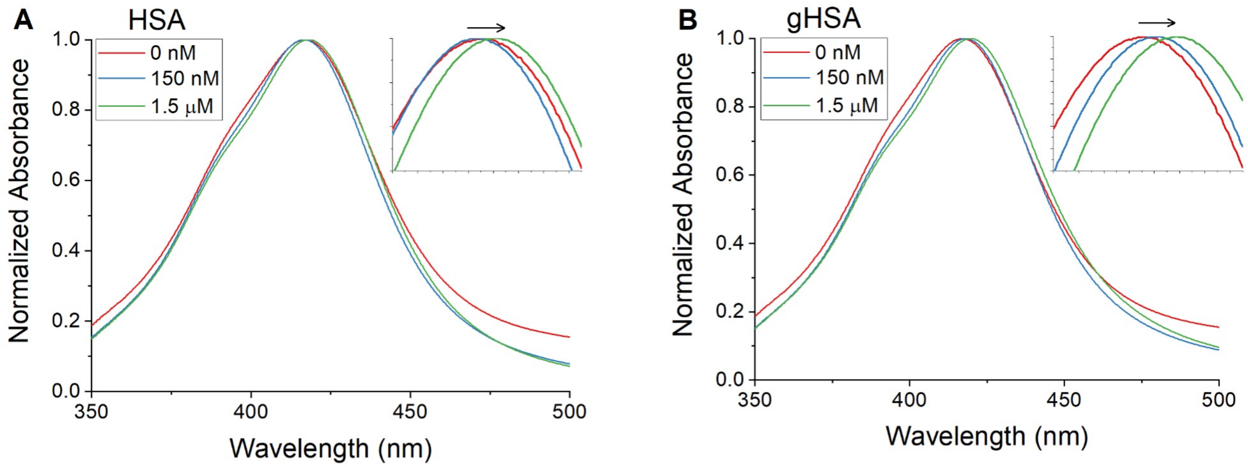

Figure S2. UV-vis spectra of AgNPs demonstrating the shift in the LSPR band with increasing concentration of **(A)** HSA and **(B)** gHSA. AgNPs were prepared to a concentration of 24 pM and the protein concentrations were as indicated. All solutions were prepared in 5 mM citrate – 5 mM NaCl buffer (pH 6.5).

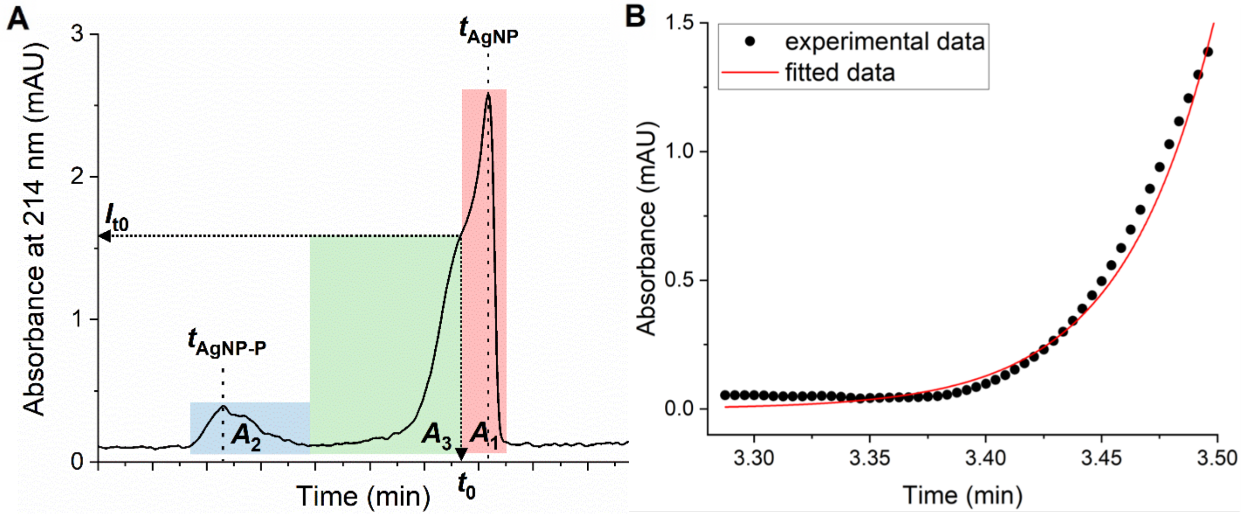

Figure S3. (A) Annotated CE electropherogram demonstrating the determination of the parameters used to calculate association and rate constants (K_d, k_{on}, and k_{off}) according to Eqns. 2-5 and in accordance with NECEEM theory. **(B)** Region A_3 of a representative CE electropherogram fit using Eqn. 4 to determine k_{off}. In these representative electropherograms, AgNPs were prepared to a concentration of 240 pM and the HSA concentration was 150 nM. All solutions were prepared in 5 mM Tris – 500 mM Gly buffer (pH 7.8).

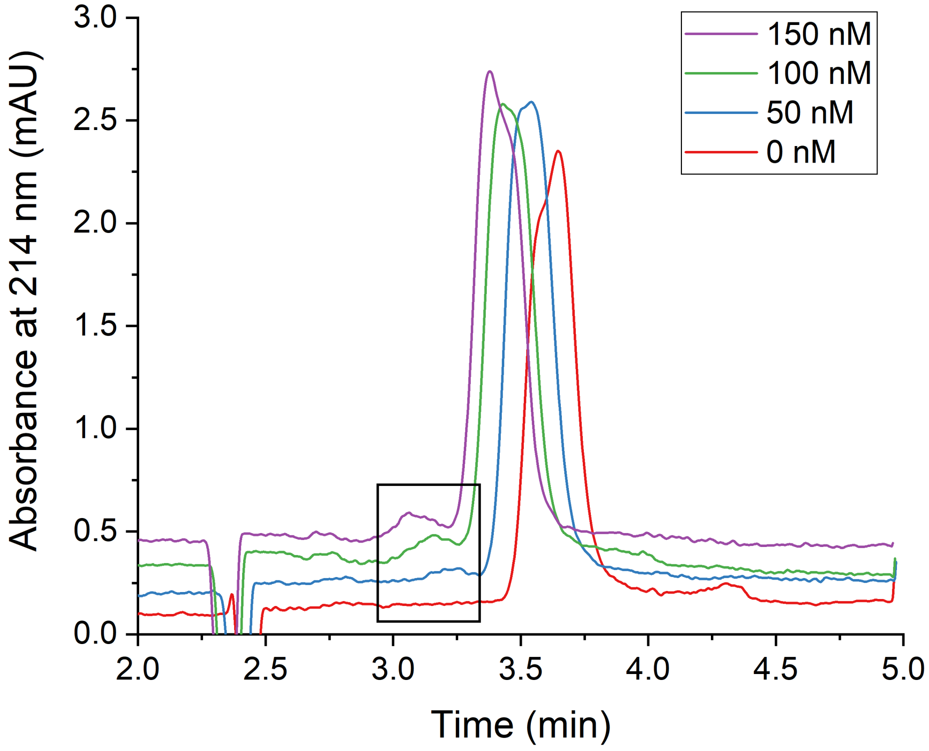

Figure S4. Representative CE electropherograms of AgNPs in the presence of increasing concentrations of gHSA. The boxed region highlights the AgNP-gHSA complex peak which increases with increasing [gHSA]. Electropherograms are vertically offset for clarity. AgNPs were prepared to a concentration of 240 pM and the protein concentrations were as indicated. All solutions were prepared in 5 mM Tris – 500 mM Gly buffer (pH 7.8).

Table S2. Average percent cell viability of HepG2 cells after 24 and 72h exposure to HSA and gHSA[a]

Protein	[Protein] (μM)	Cell Viability (%)[b]	
		24h	72h
HSA	0.703	107 ± 8	106 ± 5
	7.03	103 ± 9	98 ± 4
gHSA	0.703	99 ± 9	103 ± 7
	7.03	104 ± 10	105 ± 6

[a]Cell viability data are for HepG2 cells incubated in cell culture media containing the indicated amount of protein (no AgNPs). Data are normalized against HepG2 cells incubated in culture media alone (no protein and no AgNPs).
[b]Values represent the average and standard deviation of three samples.

Table S3. Average percent cell viability of HepG2 cells after 24 and 72h exposure to HSA- and gHSA-coated AgNPs[a]

AgNP sample	[AgNPs] (pM)	Cell Viability (%)[b]	
		24h	72h
AgNPs	10	98 ± 10	106 ± 20
	100	104 ± 8	94 ± 10
AgNPs-HSA	10	86 ± 10	71 ± 10
	100	59 ± 8	47 ± 8
AgNPs-gHSA	10	91 ± 10	74 ± 9
	100	56 ± 6	44 ± 5

[a]Cell viability data are for HepG2 cells incubated in cell culture media containing the indicated concentration of HSA- or gHSA-coated AgNPs. Data are normalized against HepG2 cells incubated in culture media alone (no protein and no AgNPs).
[b]Values represent the average and standard deviation of three samples.

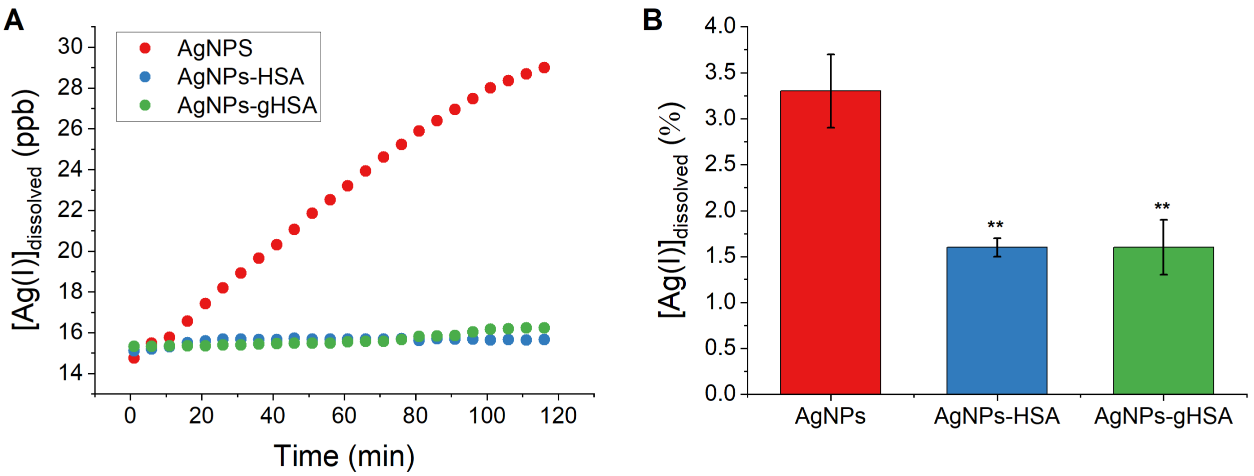

Figure S5. (A) Representative dissolution curves of AgNPs, AgNPs-HSA, and AgNPs-gHSA measured by LSSV. AgNPs were prepared to a concentration of 4.8 pM and proteins were prepared to a concentration of 300 nM. All solutions were prepared in 5 mM citrate – 5 mM NaCl buffer (pH 6.5). **(B)** Percent of dissolved Ag(I) measured by LSSV. Statistical significance was independently evaluated for each protein condition relative to the no protein control using a two-tailed *t*-test evaluated at the 99%(**) confidence interval.

Table S4. Characterization of initial AgNP dissolution rates in the presence of HSA and gHSA[a]

sample	$k_{dissolution}$ ($\times 10^{-4}$ min^{-1})[b]	% AgNPs$_{dissolved}$[c]	LOD (μM)
no protein	1.6 ± 0.3	3.3 ± 0.4	0.10
HSA	NM	1.6 ± 0.1	0.037
gHSA	NM	1.6 ± 0.3	0.083

[a]Experimental conditions were as reported in Figure S5.
[b]AgNP dissolution rate constants, $k_{dissolution}$, were calculated from LSSV voltammograms using Eqn. 8. In the presence of HSA and gHSA, the $[Ag(I)]_{dissolved}$ was nearly constant over the 2h analysis period, so the rate constant could not be reliably measured (NM).
[c]The $\%Ag(I)_{dissolved}$ was determined using Eqn. 9 from LSSV voltammograms.

Table S5. Characterization of AgNP dissolution after 24 and 72h exposure to HSA and gHSA[a]

sample	exposure time (h)	$[Ag(I)]_{dissolved}$ (%)[b]	LOD (μM)
no protein	24	5 ± 2	0.27
	72	6.5 ± 0.5	0.34
HSA	24	0.70 ± 0.05	0.22
	72	0.9 ± 0.3	0.24
gHSA	24	0.95 ± 0.09	0.10
	72	1.5 ± 0.5	0.15

[a]Experimental conditions were as reported in Figure 6.
[b]The $\%Ag(I)_{dissolved}$ was determined using Eqn. 9 from LSSV voltammograms.
